# Advancing the understanding of forest conservation dynamics through livelihood and landscape change scenarios: a case study in Chiapas, Mexico

**DOI:** 10.1007/s10668-023-02965-z

**Published:** 2023-02-24

**Authors:** Diana Alfonso-Bécares, Mario Giampietro, Esteve Corbera, Tarik Serrano-Tovar

**Affiliations:** 1 ICTA-UAB, Institut de Ciència i Tecnologia Ambientals, Universitat Autònoma de Barcelona, UAB Campus, Edifici Z, Bellaterra, 08193 Barcelona, Spain; 2 ICREA, Institució Catalana de Recerca i Estudis Avançats, Pg. Lluís Companys 23, 08010 Barcelona, Spain; 3 Department of Geography, Universitat Autònoma de Barcelona , UAB Campus, Bellaterra, 08193 Barcelona, Spain; 4 IERMB, Institut dʹEstudis Regionals i Metropolitans de Barcelona, UAB Campus, Bellaterra, 08193 Barcelona, Spain

**Keywords:** Forest conservation, Archetypes, Conservation policy, Livelihoods, Scenarios, Rural systems

## Abstract

Forest conversion and conservation in rural settings are linked to both social and biophysical drivers. However, the joint analysis of these drivers presents methodological challenges. To address this problem, we propose a novel methodology to explore the relationship between livelihood heterogeneity and land use change at the community level. It combines the concept of archetype with the accounting scheme of MUlti-Scale Integrated Analysis of Societal and Ecosystem Metabolism to define and quantify the characteristics of livelihood typologies in socioeconomic (time use, financial flows) and ecological terms (land use, agricultural inputs, soil degradation). Conservation trade-offs of potential policies are explored through “what if” scenarios assuming changes in off-farm opportunities, population growth, and conservation/farming subsidies. The approach is tested with a case study of the community of San Isidro, in Chiapas, Mexico. We conclude that the concept of livelihood typologies is useful to inform the debate over conservation prospects in rural environments.

**Supplementary Information:**

The online version contains supplementary material available at 10.1007/s10668-023-02965-z.

## Introduction

Forest conservation is interlinked to forest conversion in social-ecological systems (Ostrom, 2009). While the main identified cause of forest conversion is to clear space for agriculture (Angelsen & Kaimowitz, 1999), there is not an all-encompassing explanation as to why, where forest areas exist, productive areas expand into forest in some areas but not in others (DeFries & Rosenzweig, 2010). This calls for a better understanding of the relationship between forests, land use change and agricultural dynamics (Geoghegan et al., 2001).

Agricultural dynamics have an important role in rural livelihoods (Babigumira et al., 2014; Ellis, 1998; Scoones, 2015). For example, economists have pointed to the existence of poverty traps linked to initial household endowments in land and assets, with small land holdings limiting livelihood prospects for farmers and conditioning land expansion (Barrett & Carter, 2013; Coomes et al., 2011; Scheidel et al., 2014). This affirms that social distribution impacts forest clearing and land use change (Coomes et al., 2016). Dorward et al (2009) have identified three main livelihood strategies among the complex array of paths: (1) “stepping up” or investing in agricultural activities to increase production; (2) “stepping out” or investing in assets for high return in other, usually off-farm activities; and (3) “hanging in” or engaging in activities that allow to maintain minimum livelihood levels (Dorward et al., 2009). This classification underpins the different constraints under which households operate with regard to land use (Dobler-Morales et al., 2022). Although land use models have been used to explore the effects of possible future changes in forest cover (Angelsen, 2010; Hersperger et al., 2010; Van Khuc et al., 2020), the effects of social drivers, such as the initial landholding, have only been analyzed in isolation on account of the methodological challenges to integrate both aspects (Coomes et al., 2004, 2011; Norgaard, 2008).

Given the entanglement of land use and human welfare (Geist & Lambin, 2002; Kaimowitz & Angelsen, 1998; López-Carr, 2021; Tenza et al., 2019), some conservation policies have financially incentivized rural dwellers to promote forest conservation (Blundo-Canto et al., 2018; Farley & Bremer, 2017) in an effort to halt land use change while simultaneously reducing poverty. In the last decades, Payment for Ecosystem Services (PES) has been one of the main schemes following this avenue (Shapiro-Garza et al., 2020). However, while there is consensus that forest conservation should not be approached without considering livelihoods(Goh & Yanosky, 2016), the joint achievement of both objectives has proven elusive (Calvet-Mir et al., 2015; Corbera et al., 2007). This suggests that more research is needed to understand the relevant characteristics of the context where such policies unfold (Alfonso-Bécares, D. Corbera, 2020; Liu & Kontoleon, 2018; Van Hecken & Bastiaensen, 2010).

Based on these premises, in this article we analyze the relationship between livelihood heterogeneity and land use dynamics to better understand the potential drivers of forest loss as well as the potential impacts of conservation policies on livelihood strategies. We posit that the option space of farmers to engage in forest conservation is contingent on their livelihood strategy and initial land endowment. We explore this hypothesis with scenarios that simulate conservation schemes in a rural community. To analyze the entanglement between land use and livelihoods, we use a novel approach that combines the concept of archetypes (Eisenack et al., 2019; Oberlack et al., 2019) with the accounting scheme of MUlti-Scale Integrated Analysis of Societal and Ecosystem Metabolism (MuSIASEM) (Giampietro, 2003; Giampietro & Mayumi, 2000).

We illustrate our approach with data from a study on the community of San Isidro in Chiapas, southeastern Mexico, where the agricultural frontier has been rapidly expanding over the last 20 years (Berget et al., 2021). While a PES conservation program has been implemented in the collectively managed areas of this community (Alonso-Vázquez et al., 2012), deforestation continues in the individually managed areas. Since Mexico’s communities are increasingly shifting away from collectively managed to individually managed land (Lawrence et al., 2020), this case represents fertile ground to study the entanglement between land uses, livelihoods, and forest conversion and conservation.

Other applications of MuSIASEM to the analysis of farming systems are available (Arizpe et al., 2014; Gomiero & Giampietro, 2001; Pastore et al., 1999; Serrano-Tovar & Giampietro, 2014) but, to the best of our knowledge, this is the first time MuSIASEM is used to explore the links between livelihoods, land use change and forest conservation.

## Methodology

### Study area: land use and conservation programs

**Fig. 1:**
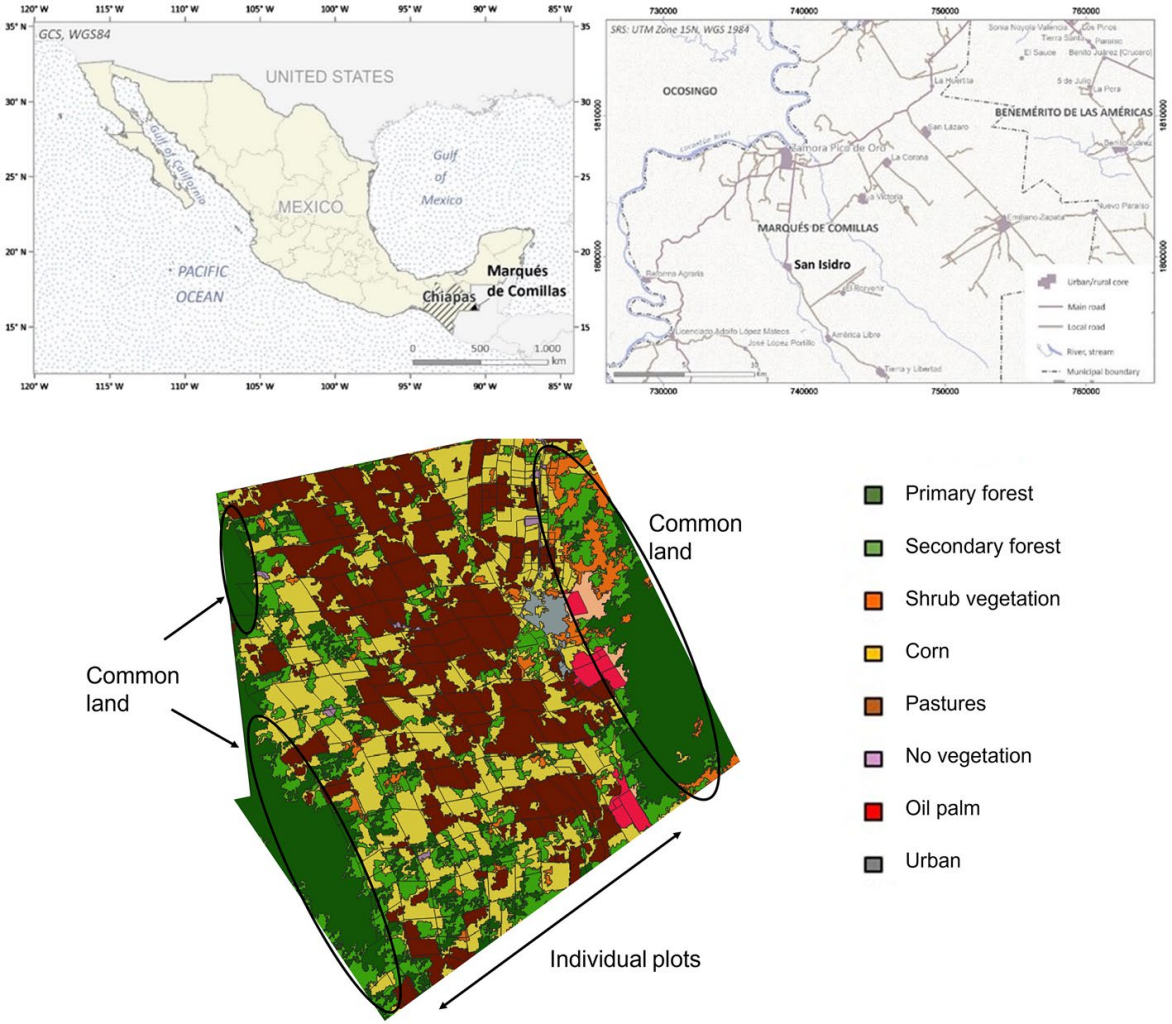
Geographical location and land cover of the community of San Isidro

The study focuses on San Isidro, a landlocked community in the municipality of Marqués de Comillas, in the state of Chiapas, Mexico (Fig. 1). Marqués de Comillas, the municipality of which San Isidro is part, has received successive waves of settlers from the 1970s onwards (Carabias et al., 2015; de Vos, 2002). These settlers organized in ejidos, i.e., a form of collective land tenure where households can farm individual plots of land while maintaining a communal holding (Appendini, 1997). The federal government frequently granted the settlers between 20 and 30 ha of land spread across the most suitable area for farming or cattle ranching, while the remaining lands—usually forests in farther and less fertile areas—remained managed in common.

Colonization in Marqués de Comillas from 1970 onwards resulted in acute deforestation and land use change (de Vos, 2002). Maize and bean production have been subsidized by the national agricultural program Procampo since 1994, and cattle ranching has been supported by the program Progan since 2004; simultaneously, cash crops such as cocoa, coffee, cardamom, rubber, chili, or oil palm have also been promoted through different public and private subsidy schemes (Carabias et al., 2015). As a result, 21,400 ha (27% of standing forests) in the municipality have been converted to pastures or fallow since the late 1990s, while in San Isidro in particular, 36% of forests (1425 ha) have been converted between 2001 and 2018 (Hansen et al., 2013).

PES programs have been implemented in the region since the mid-2000s. In most communities where such programs have been implemented, forest conversion has been halted to some extent and forest clearing has mainly taken place in the non-protected plots (Costedoat et al., 2015). Specifically, in San Isidro, 780 of the 903 hectares of forests held in common are currently being conserved through a PES contract. The contract was issued in 2013 under the national PESL program (Special Program for the Conservation of the Lacandon Rainforest) and renewed in 2018 for another five years. Forest loss has thus mostly occurred in individual farming areas, which occupy 2560 ha of the total extension (3952 ha) of the community. As for social support, given that the community displays a high degree of marginalization (SEDESOL, 2016) a conditional cash transfer program Progresa (which now goes under the names Oportunidades and Prospera) has been in place since 1997, which has incentivized school attendance and health check-ups (Izquierdo-Tort et al., 2019).

### The conceptual framework

Given that economic land use (change) models are generally built at the micro (household) level, they only apply to a very small scale (Kaimowitz & Angelsen, 1998). For this reason, we developed a novel approach to describe livelihood typologies whose characteristics can be scaled up—by considering their relative weights—to the community or landscape level. To this purpose, we adopted the concept of archetype as an intermediate level of abstraction to identify groups of households sharing a same set of farming system characteristics. Hence, we considered archetypes as “representative patterns which advance comparison and generalization at an intermediate level in between the particularities of single cases and panacea perspectives” (Sietz et al., 2019). To integrate the demographic and socioeconomic characteristics of households with the biophysical characteristics of their land use, we used the accounting scheme of MuSIASEM. In this way, we obtained a description of the paces of the flows of biomass and economic resources in relation to both the time use (human activity) and the land use for each livelihood type.

The quantitative characterization of livelihood types was then used to analyze the relation between livelihood heterogeneity (at the household level) and overall land use change at the community level. Changes within the different livelihood types as well as in their relative contribution (relative landholding size of each livelihood type) are expected to generate changes in land use at the community level. To explore this mechanism, we built scenarios that assume changes in the total population of the community (affecting the available land per capita) and/or in economic variables (affecting the economic return of the different farming strategies). For each scenario we analyzed the impact of these changes on the distribution of households among the livelihood types, the extent of forest conservation at the level of the community, and poverty reduction at both the level of individual livelihood types and at community level.

### Accounting scheme: MuSIASEM

MuSIASEM provides an accounting scheme for quantifying the metabolism of social-ecological systems (Giampietro et al., 2021). The scheme establishes quantitative relations between the hierarchical levels of a system in two dimensions of analysis: socioeconomic and ecological (biophysical). In our study, we considered two main hierarchical levels (defined in terms of either administrative unit or space): individual households (connected to farms) and community (connected to landscape). The concept of archetypes allows to establish a bridge among these two levels.

MuSIASEM builds on the flow-fund model of (Georgescu-Roegen, 1975), which was specifically developed to study the biophysical roots of the economic process. Funds are elements that describe “what the system is,” such as human activity and land use, while flows are elements that describe “what the system does,” such as the throughput of biomass, water, and money. Flows enter the system as inputs and leave the system as outputs, becoming either wastes or useful products, whereas funds remain unaltered for the duration of the analysis and permit the metabolic conversion of the flows (Giampietro et al., 2021). Flow-fund relations are described through intensive variables, either in relation to time (i.e., the pace of flows, such as the income generated per hour of human activity) or space (i.e., the density of flows, such as cereal yield per hectare of land), and define the metabolic pattern of the system. The size of the system is described by extensive variables referring to the size of the funds (land, human activity).

**Fig. 2:**
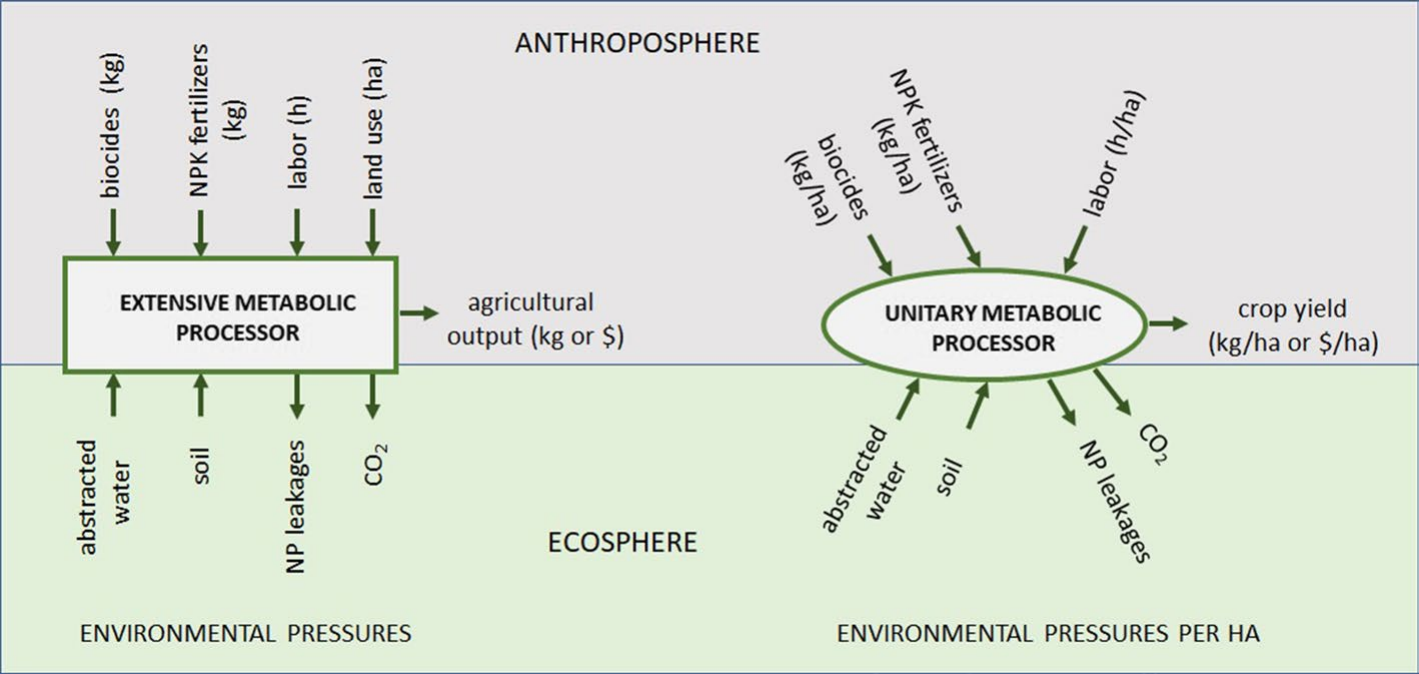
An example of an extensive (left) and unitary (right) processor describing the metabolic profile of land use

‘Metabolic processors’ are used in MuSIASEM to represent the inputs and outputs associated with a metabolic unit (e.g., a plot of land, a household, a community) (see Fig. 2). They connect flows (e.g., agricultural inputs, agricultural produce, and money) to fund elements (land, labor), thus allowing the analyst to describe the input-output profile of a metabolic unit in both extensive terms (through the size of funds) and intensive terms (through the flow/fund ratios). Figure 2 provides a schematic representation of an extensive processor (left graph) and an intensive or unitary processor (right graph). The former refers to the metabolic profile of land use at the community level expressed as a set of relations over extensive variables, i.e., the actual, observed values of inputs and outputs. The latter refers to the metabolic profile of a given land use (e.g., cereal cultivation) expressed in terms of intensive variables, in this case, per unit (ha) of land use.

The relation between: (i) the size of the fund elements (the agent that is either consuming or producing a flow) and (ii) the pace and density of processing of the metabolized flows per unit of fund size, allows the analyst to scale the characteristics of metabolic units across hierarchical levels of analysis. Thus, the possibility of bridging dimensions of analysis and scaling allows the analyst to integrate quantitative information coming from different descriptive domains and different levels of aggregation and, hence, adds a quantitative dimension to the concept of livelihood archetypes.

More details on the conceptual foundation of MuSIASEM are available in (Giampietro et al., 2011, 2021).

### Data collection

The data used in the analysis stem from a combination of first-hand fieldwork information about agricultural practices and prices, obtained by the first author during two visits to the community of San Isidro in 2018 and early 2020, local records on land uses and agricultural production for the municipality of Marqués de Comillas to which San Isidro belongs (used as benchmarks), spatial analysis, and information found in the literature (Alonso-Vázquez et al., 2012; Carabias et al., 2015; González Ponciano, 1991). Two members of the biodiversity conservation NGO Ambio, an elected authority of San Isidro, and eleven farmers of the community were interviewed. The interviews with the farmers and local experts were used to fine-tune our estimations (e.g., for yields, fertilizer and pesticide use and soil degradation). Because of the Covid-19 pandemic, a more exhaustive dataset could not be obtained.

Spatial data include land cover data for the years 2015 and 2019 collected by the Laboratorio de Análisis de Información Geográfica y Estadística (LAIGE), using SPOT 5 (10 m pixel units). Spatial data of forest loss were obtained from Global Forest Watch (Hansen et al., 2013). The spatial division between individual and communal plots was extracted from the Mexican Agricultural Census of 2016 (INEGI, 2016) and the land tenure data (the distribution of the individual plots) from the cadastral map of San Isidro drawn in 2006 for PROCEDE.^1^ These data were processed using GIS software ArcMap 10.7.1 and QGIS 3.4.7. to obtain the land endowment and the different land covers associated with the plot(s) of each *ejidatario*. Demographic and agricultural data were obtained from official documents and censuses (INEGI, 2010, 2015, 2020; RAN, 2010; SIAP, 2016).

### Construction of livelihood types

**Fig. 3:**
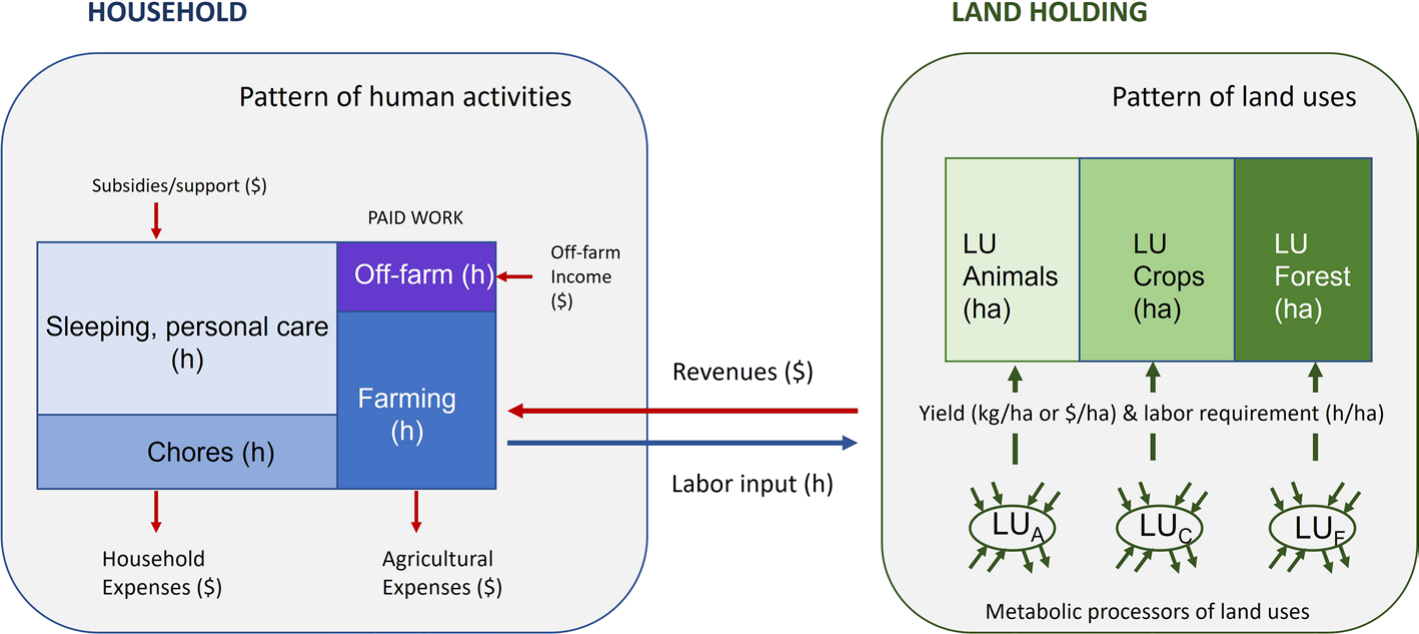
Characterization of the basic metabolic unit, the household, in terms of time use (left box) and land use (right box)

In our application of MuSIASEM, the household is the basic metabolic unit expressing an integrated set of on-farm, off-farm, and non-farm activities (required for its survival and reproduction). This is schematized in Fig. 3 for San Isidro, a community that relies for its livelihood on a diverse set of land uses (LU), namely pasture for livestock rearing, crop cultivation and forest areas, as well as off-farm and non-farm activities. By combining the metabolic characteristics of the set of activities (time use) of the household (left-hand side of Fig. 3) with those of the associated land uses (right-hand side of Fig. 3), we obtain the metabolic characteristics (socioeconomic and biophysical) of the household's livelihoods.

As there were very few off-farm opportunities in San Isidro, we defined the baseline livelihood typologies based on the land use information for each household for the year 2015, using as the differentiating criterion the main land use (in terms of surface) in their landholding. In this way, we sorted the households into 3 different livelihood types, corn (Ct), ranching (Rt), or forest types (Ft), depending on the main farming orientation. However, if the difference between the primary and the secondary land use of the household was less than 3 hectares, the household was assigned to a fourth typology, namely “mixed strategy” (Mt).

To obtain a quantitative characterization of the four livelihood types, we first assessed the expected metabolic characteristics of the main agricultural production processes in the area (corn production, cattle rearing, palm production), using data collected at the municipal level (the municipality of Marqués de Comillas), including the volume of the crops produced (output), the areas cultivated to the various crops, and the market prices in the region (INEGI, 2016; SIAP, 2016). These data were used to calculate benchmarks defining the expected yield and income per hectare (intensive variables) for each land use. These benchmarks were checked for congruence against the information collected during the field work and regionally based information from the literature (Alonso-Vázquez et al., 2012; Carabias et al., 2015; González Ponciano, 1991). The (initial) landholdings of the households of San Isidro were obtained from the local cadaster. Integrating the information on land holdings with the assessment of income per hectare allowed us to calculate the on-farm income of different typologies of households. This allowed to obtain information on household monetary flows associated with agricultural production, that was complemented with locally gathered information on social and agricultural subsidies as income sources.

Land use changes in San Isidro were assessed using satellite data on land use for 2015 and 2019. By using the cadastral references of the plots undergoing change, we assessed land use change at the household level, and subsequently scaled these changes up to the archetype level. Note that in our scenario analysis, changes in livelihood strategy at the household level occur if the household's main land use changes or if the difference between the primary and secondary land use crosses the cut-off point of 3 ha (in either direction). This switching among livelihood types at the household level changes the relative distribution (and weights) of livelihood types at the community level. The economic, institutional, and biophysical conditions playing a role in the decisions about land conversion and changes in livelihood strategies at the household level were reconstructed from the information collected through the interviews with farmers and information from the literature.

### Scenarios

Using the four livelihood types defined in the previous section, we built “what if” scenarios to explore possible futures of the community in terms of land use and income. In all scenarios considered, the simulation period is 12 years, from 2019 to 2031**.** Three scenarios were considered:

1. *Scenario 1*: Present trend. We assumed that land conversion to livestock pastures continues to follow the dynamics of land use change observed between 2015 and 2019, including the switching between livelihood types (see Sect. 3.2). Note that the same observed four-year trend was iterated 3 times. We further assumed a population growth rate of 4.6% per year (based on the average growth rate in the period 2015 and 2020 in the village). Given that there is no room for expansion of the current farming land in San Isidro, we assumed that the number of landholdings and households remains constant in time. Hence, we assumed that population growth increases household size, but not the number of households. This scenario is compatible with the livelihood strategy “hanging in” of (Dorward et al., 2009).
2. *Scenario 2*: Off-farm jobs. In this scenario, we assumed that a new policy implemented by the central government will generate local sources of non-specialized off-farm jobs in the area in 2022, which will allow households to change their main livelihood strategy. This scenario is compatible with the livelihood strategy “stepping out” of (Dorward et al., 2009).
3. *Scenario 3*: Stronger forest conservation policy. In addition to the traditional PES payment in the common areas (1000 MXN/ha or 53 USD/ha),^2^ a subsidy of 6000 MXN/ha (317 USD/ha) is paid by the central government to incentivize conservation of forest plots in individually held areas vulnerable to land conversion if the present trend was followed. This subsidy is granted to farms where forest areas represent more than 10% of the total landholding, so Rt livelihood type would be excluded. This scenario serves to examine the impact of public policies promoting forest conservation on individually held land.

For scenarios 2 and 3, we considered two variants that differ regarding the assumptions about population growth, i.e., availability of natural resources per capita. In the first variant, population growth is assumed to continue at the current rate of 4.6% (same assumptions as in scenario 1). In the second, the population is assumed to stagnate from 2019 onwards, and the number of members per household as well as the total number of households remains the same from 2019 until 2031.

We compared the scenarios with regard to expected socioeconomic welfare and expected ecological performance in 2031. Socioeconomic welfare was approximated by income per capita, and ecological performance was evaluated through three indicators: change in the size of forests, level of chemical input, and soil degradation status.

For scenario 3, we calculated the sum of social and agricultural subsidies (Prospera, Procampo and Progan) in each livelihood type to infer the net cost of this new policy if the traditional agricultural subsidies were dropped (see table TS6 for details).

## Results

### Characterization of the livelihood types

The analysis of initial landholdings and land use for 2015 showed that most of the 119 households in San Isidro tended to converge to a single type of farming activity—either corn cultivation, cattle ranching, or forest conservation. Households with a mixed farming profile were considered as a separate group. Hence, the following four livelihood types were considered:

1. Corn oriented or milpa type (Ct) in which the main activity is the traditional milpa system, combining corn and beans, with a mean surface of 10.5 ha, and smaller surfaces of pastures for bovine rearing and forests (around 3 ha each).
2. Ranching oriented type (Rt) in which the main activity is bovine rearing (average area of 17 ha and approximately 2 cows/ha (SIAP, 2016), with a smaller area dedicated to milpa and forest (6 and 2 ha, respectively).
3. Mixed strategy type (Mt) in which similar areas of the holding are allocated to milpa and ranching (8 ha each) and a smaller surface to forests (6.5 ha).
4. Forest oriented type (Ft) in which the larger surface of the holding (11 ha) is covered by primary and secondary forests and a small surface dedicated to milpa (4.5 ha) and in which the household (partially) relies on off-farm sources of income.

**Fig. 4:**
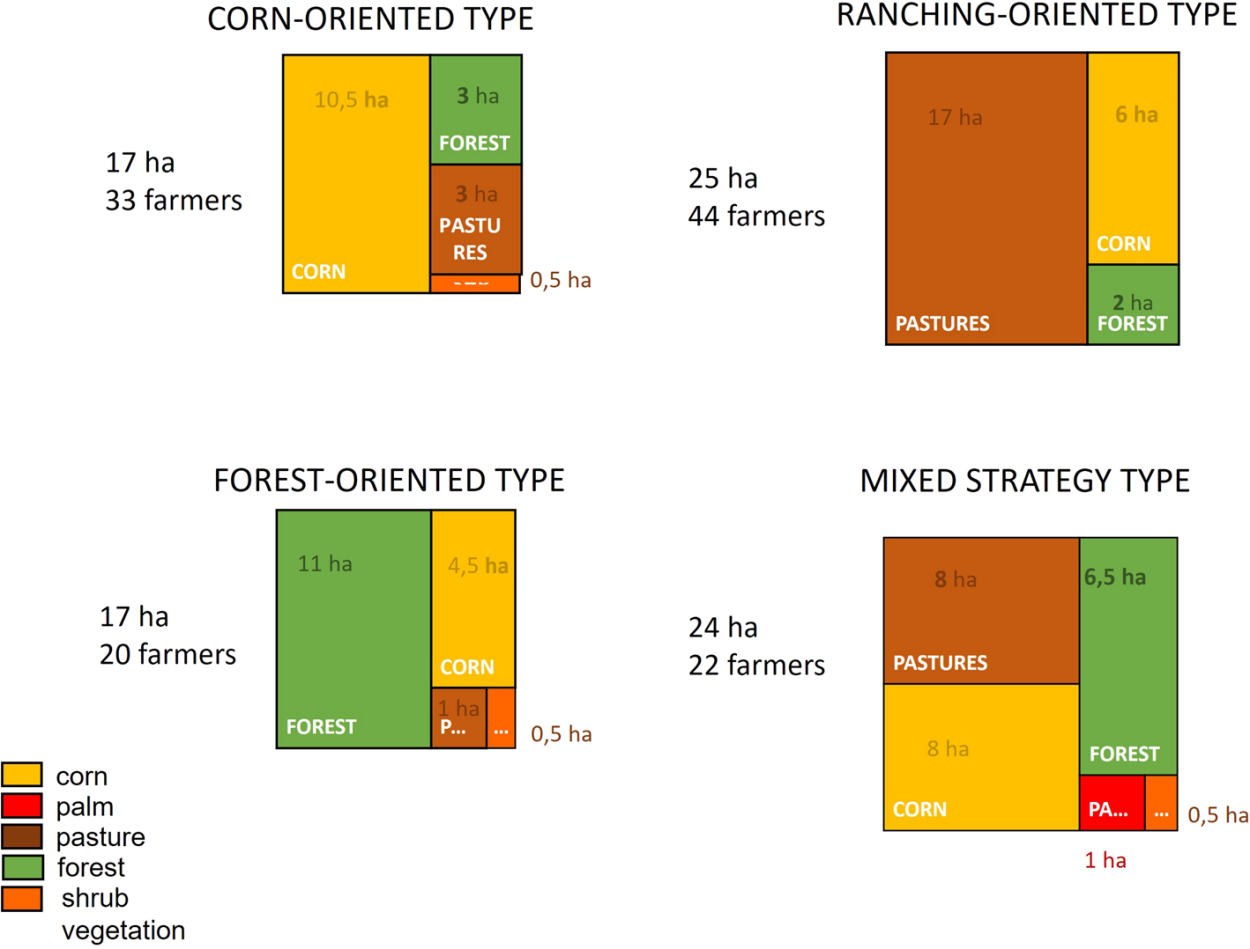
Distribution of the households of San Isidro among the four livelihood types

The distribution of the 119 households in the community according to these types is shown in Fig. 4. Forty-four (37%) of the households belong to the Rt, 33 households (28%) to the Ct, 20 (17%) to the Ft and 22 (18%) to the Mt. The larger farm surfaces belonged to the Rt and Mt types, with a mean of, respectively, 25 and 24 ha. Details on the agricultural production (output, land area and yield) and income for each livelihood type are reported in Tables TS1 to TS5 in Supplementary Material.

In 2015, the mean agricultural income per capita and year was 16,000 MXN (1068 USD^3^) for the corn type households, 72,000 MXN (4809 USD) for the ranching type, 37,000 MXN (2471 USD) for the mixed strategy type and 7000 MXN (468 USD) for the forest type. These values were used as a baseline in each of the scenarios analyzed. Given that the average per capita income per year of the forest livelihood seemed too low to ensure living conditions, we assumed the existence of additional income from either remittances or off-farm jobs for this livelihood type. In fact, the extensive cattle rearing practiced in the area is less labor intensive (per hectare) than corn production (SAGARPA, 2015) which permits off-farm work. An additional income of 70,000 MXN (3703 USD) per household per year was assumed. This amount is equal to the minimum wage for an agricultural worker in 2022 (CONASAMI, 2022). This adjustment increased the per capita income for the forest type households to 19,000 MXN (1005 USD) per year. Considering the distribution of households among the livelihood types, the mean yearly income per capita in the San Isidro community in 2015 was 41,000 MXN (2169 USD) (weighted average).

### Observed land use dynamics between 2015 and 2019

**Fig. 5:**
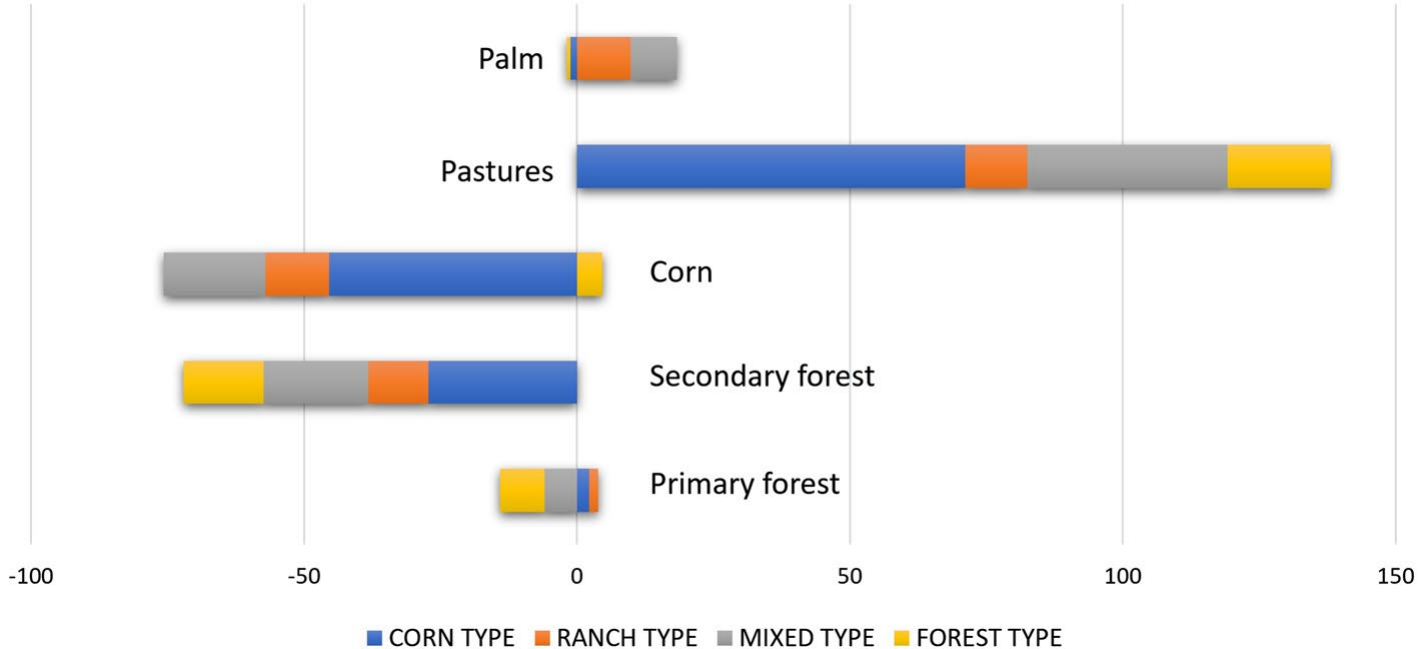
Contribution of livelihood types to land use changes observed in San Isidro between 2015 and 2019. Changes in area are reported in ha

The observed land use changes in San Isidro over the period 2015 and 2019 served as the baseline trend to construct the scenarios. Observed land use changes at the household level were connected to livelihood types (see Sect. 2.5). This allowed us to assess the contribution of each of the livelihood types to the observed land conversion at the community level (Fig. 5). Note that the total area allocated to individual farming in San Isidro (2560 ha) remained unchanged between 2015 and 2019. As shown in Fig. 5, the corn livelihood type (Ct) accounted for most of the land use conversion, by switching from traditional milpa cultivation (and to a lesser extent, secondary forest) to pastures.

Land use changes in San Isidro between 2015 and 2019 can be attributed to changes in the relative composition of livelihood types as well as (smaller) land use conversions within each of the livelihood types (households converting part of their land but not in a sufficient degree to change classification; see Fig. S1 in Supplementary Material). As for the former, three households originally belonging to the corn livelihood type (Ct) switched to ranching (Rt) and five to the mixed strategy (Mt). Seven of the households originally classified as mixed strategy (Mt) switched to ranching (Rt), and four households from the forest type (Ft) to the mixed livelihood (Mt). The strongest attractor (the livelihood to which other types tend to switch most) thus appeared to be ranching, followed by the mixed livelihood. Changes observed inside the livelihood types followed the same trend, i.e., an increase in the surface dedicated to pastures compensated by a reduction in the areas dedicated to forests, corn (milpa), or both. Even within the ranching livelihood type, the strongest attractor and the livelihood already accounting for most of the land use in 2015, pastures plots were expanded in the period 2015–2019 and forest and corn plots reduced.

The economic, institutional, and biophysical conditions of San Isidro play an important role in the households’ decisions about land conversion. As for the economic conditions that influence land conversion, subsidies to corn cultivation amounted to between 770 and 1000 MXN (40.75 and 53 USD) per hectare per year in 2017, whereas the subsidies to cattle ranching were 600 MXN (31.75 USD) per hectare that same year. The PES program in San Isidro rewards the community with 1000 MXN for each hectare registered under the conservation contract. The community registered 780 hectares during two consecutive contract periods: 2014–2018 and 2019–2023.

Required conversion investments for cattle ranching and corn cultivation differ markedly. While the investment needed to prepare a field for corn production is approximately 4300 MXN/ha (227.5 USD/ha), cattle ranching needs an initial investment of about 55,000 MXN/ha (2910 USD/ha) to purchase animals and infrastructure (Carabias et al., 2015). However, the marginal labor needed to maintain the infrastructure and tend to the cattle decreases for every additional hectare and so does the marginal monetary input. In contrast, corn cultivation does not benefit much of scale economies (in terms of either monetary input or labor volume) when the area cultivated is expanded. This is because corn cultivation still follows the milpa tradition and is mainly unmechanized and rainfed (no irrigation).

**Fig. 6:**
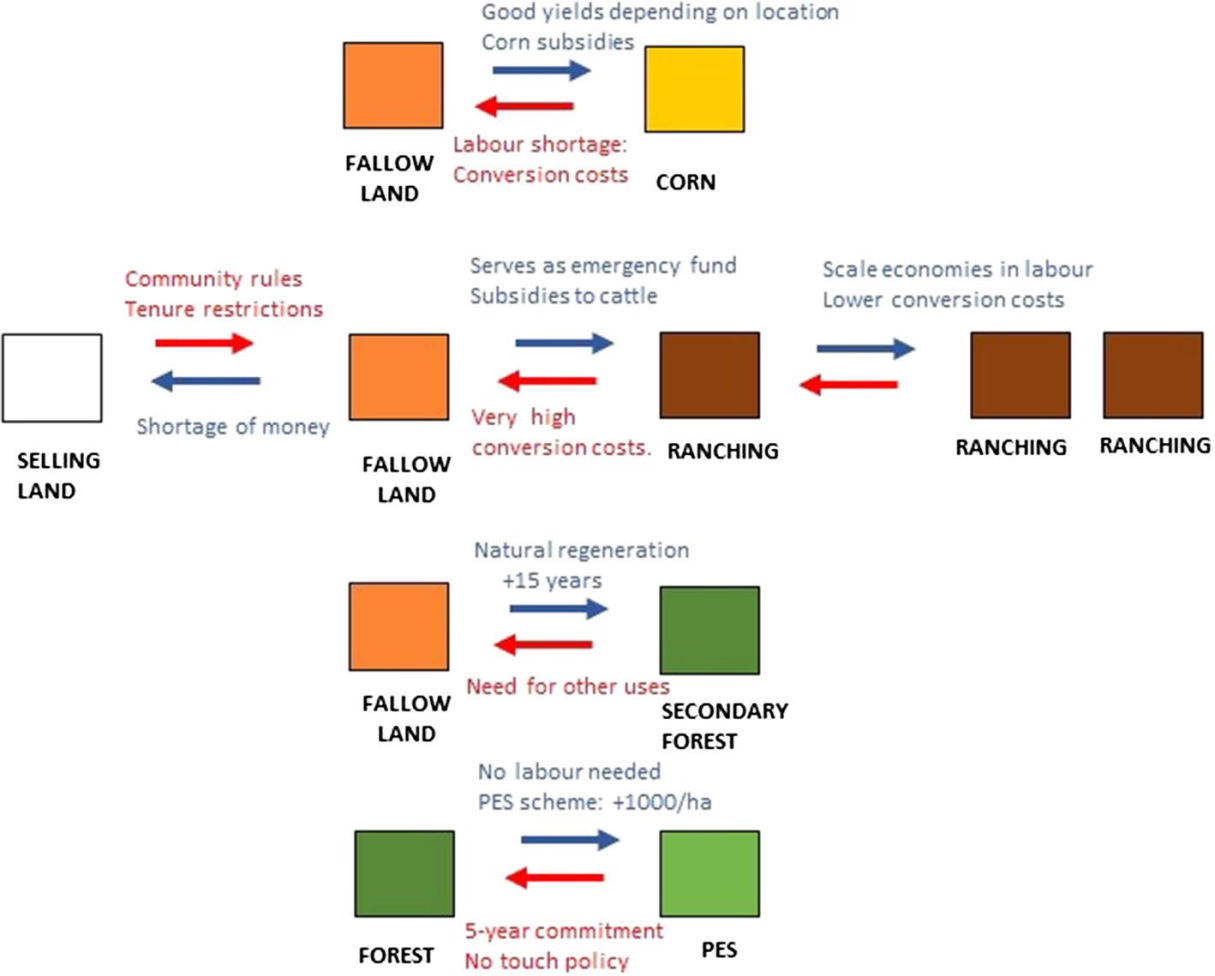
Incentives (blue arrows) and obstacles (red arrows) to land use change in the study area (PES = payment for ecosystem services)

Based on these insights, we summarize the major incentives and obstacles that might influence the option space of farmers in Fig. 6. Other relevant factors (not shown) affecting livelihood choices are that the remote location of San Isidro does not favor the integration of agricultural products in the regional or national markets and that bare soil is especially prone to hydraulic erosion in the area (Myers 1988) both discouraging corn production, hence the continuity of Ct types.

### Results of the simulation

#### Scenario 1: present trend

**Fig. 7:**
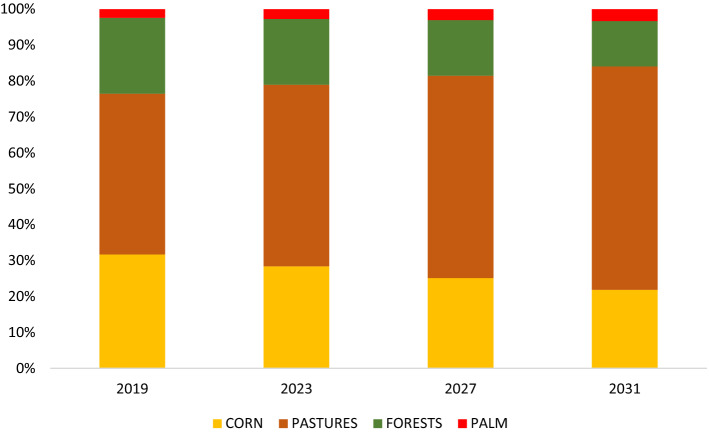
Expected land use change (% of surface of individual farming land) from 2019 to 2031 in the present trend scenario (scenario 1). Indicators are detailed in Table TS3 of the supplementary material

The expected land conversion in San Isidro in the present trend scenario is illustrated in Fig. 7. This figure shows that pastures would progressively take over the landscape in the period 2019–2031, eventually covering 62% of the individual farming land in 2031, with corn and forests occupying 22% and 13%, respectively. In this scenario, in 2031 less than 1% of the households would belong to the livelihood type Ct; 3% to Ft, 26% to Mt, and 70% of households to Rt.

**Fig. 8:**
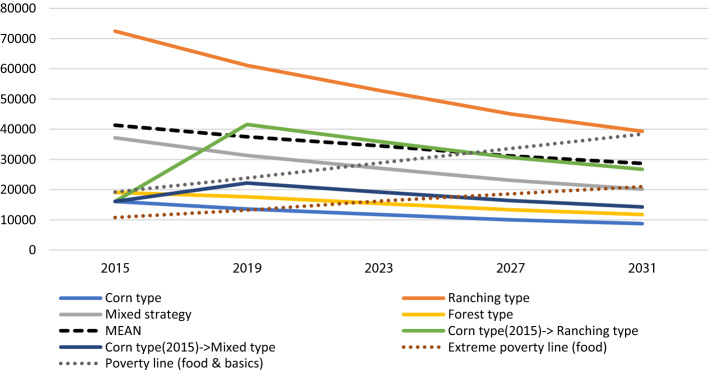
Expected changes in income (in MXN) per capita per year for the different livelihood types in San Isidro in scenario 1 (present trend)

Figure 8 shows the expected changes in income (in MXN) per capita per year for the different livelihood types in San Isidro. With the total area allocated to individual farming in San Isidro remaining unchanged, and population growth continuing the rate observed between 2015 and 2019, economic conditions would worsen for all four livelihood types. While Rt would remain the livelihood type with the highest per capita income in this scenario, the households that switch from Ct to Rt during the scenario period would not reach as high an income as the households that were already classified as Rt at baseline situation because of their smaller land endowment.

Also shown in Fig. 8 is the minimum yearly budget by which means a person can be provided with food (extreme poverty line) or with food plus other basic products (poverty line). These indicators of socioeconomic welfare are defined by the Mexican Agency Secretaría del Bienestar (CONEVAL, 2021; SEDESOL, 2016) and have been extended to future years by projecting the trend observed between 2016 and 2021 (an increase of 750 MXN (40 USD)/month for the extreme poverty line and 1250 MXN (60 USD)/month for the poverty line). As can be seen in Fig. 8, in the present trend scenario, only the Rt livelihood types (those originally classified as such as well as those who changed to this livelihood during the scenario period) would reach an income per capita above the extreme poverty line.

#### Scenario 2: off-farm jobs

**Fig. 9:**
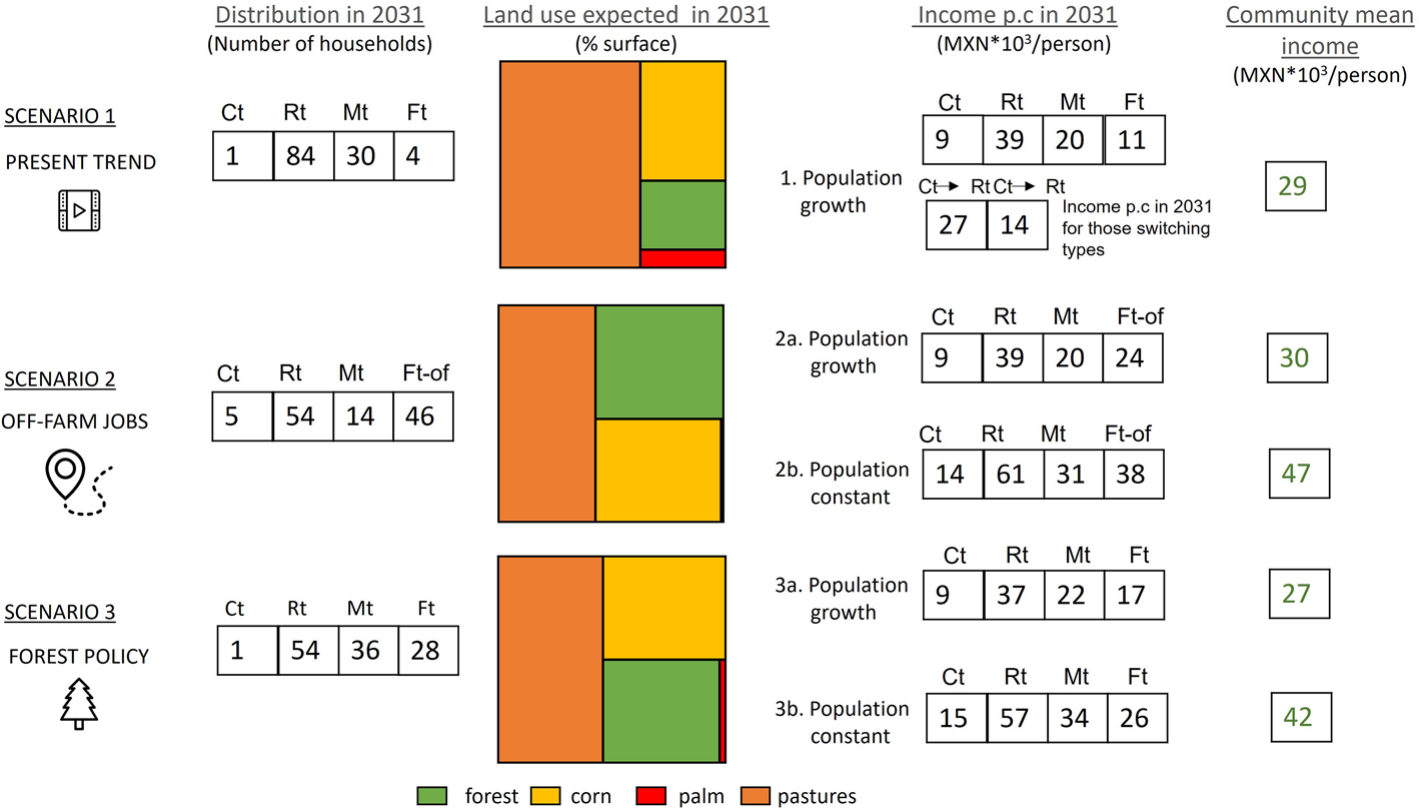
Distribution of livelihood types (in number of households), land uses (in %), and income per capita (in thousands MXN per year) in San Isidro in 2031 in the three scenarios considered

In this scenario, we tested whether policies reducing the dependence of rural livelihoods on land-based activities would improve the income in the community and reduce the pressure on the land. We assumed that two workers per household would be able to engage in off-farm employment together contributing 160,000 MXN (8465 USD) per household per year. With this new availability, the Ft type have better economic prospects. We expect then that households belonging to other livelihood types would now have a full-time job outside the farm, emulating the land use of the Ft. This Ft-off livelihood type takes full advantage of the off-farm jobs and as a result prioritizes forest surface over other land uses. The results of this scenario are illustrated in Fig. 9.

Figure 9 suggests that the resulting land use changes in this scenario could help mitigate the deforestation anticipated in the first scenario (present trend). The number of households belonging to the more “environmentally friendly” Ft-off-farm livelihood type would increase gradually, reaching 46 households in 2031, with the arrival of both former Ct and Mt households (Fig. 9). The areas dedicated to pastures would be reduced to 43% in 2031, forest would cover 30% of the individual farming land and corn 26%.

If population growth would continue its present trend (scenario 2a in Fig. 9), the mean per capita income per year would slightly decrease over the years, despite the availability of off-farm employment (see table TS2 in Supplementary Material). The reason is that the reduction of the available land per capita (due to population growth) offsets the beneficial effects of the policy. Although income prospects are better than in scenario 1, there is still no significant improvement in the average income at community level (Fig. 9). If, on the other hand, we assume no population growth and hence a constant availability of land per capita (scenario 2b in Fig. 9), the general standard of living would improve and all livelihood types, but Ct would reach an income per capita above the extreme poverty line.

#### Scenario 3: Stronger forest conservation policy

In this scenario, we assumed that a new policy would target individually held forests areas of the households belonging to livelihood types Ct, Ft and Mt, and compensate their owners to conserve standing forests thus discouraging the switching to other livelihoods linked to higher forest loss. Our scenario also assumes that the previously existing incentives to agricultural production and ranching are discontinued, and forest conservation is prioritized. We set an amount per hectare of forest of 6000 MXN/ha (317 USD/ha) to overcome the mean income obtained per hectare obtained by the Ct households (4500 MXN /ha (328 USD/ha) and to discourage the conversion of forest plots to pastures. Given the forest areas of each livelihood type, this new subsidy would amount to a mean of 18,000 MXN per household per year for Ct households, 39,000 MXN for Mt households and 66,000 MXN for Ft households. The investment would increase over the years since we expect households to progressively switch to Ft livelihood type. Table TS6 in Supplementary Material summarizes the expected investment, with the amounts of the new forest policy overcoming the expenses of the traditional agricultural policy from 2031.

Given that Rt households only have less than 10% of forest in their mix, they are excluded from the program since the relatively small incentive amount would not suffice to discourage their extensive ranching.

The results in Fig. 9 show that in this scenario the land dedicated to livestock rearing would decrease to 46% and the land dedicated to corn cultivation and forests would each occupy approximately 25% of individual land endowments. Ft and Mt types would each have 12 more households switching from Ct in 2031. The reason is that in this scenario, the Ft livelihood, having a larger forest area, gives the same income as the Ct livelihood, but with a lower workload. The Mt livelihood, dividing the land holding among corn, pastures, and forests, also provides a higher income than Ct (Fig. 9).

If the population keeps growing at the same pace as between 2015 and 2019 (scenario 3a), the standard of living would decrease for every livelihood type (Fig. 9). However, assuming a constant population size (scenario 3b), the mean income would increase, especially for the Ft and Mt livelihoods. This could possibly incentivize Ct households to shift livelihood strategy, allocating more land to forests and less to corn. The income prospects for the Rt livelihood in this scenario would be lower compared to the other two scenarios because the Rt households are not benefiting from the new conservation scheme nor receiving any longer the ranching subsidies (see table TS6).

#### Socioeconomic and ecological indicators at community level

By considering the changes in the distribution of livelihood types and the changes in their income in relation to the poverty and extreme poverty lines (shown in Fig. 9), we obtain an indication about the socioeconomic impact of the scenarios for the community of San Isidro (see Table 1). The mean per capita income in San Isidro would fall below the poverty line in all scenarios, except for scenarios 2b and 3b in which we assumed no population growth. The percentage of households falling under the extreme poverty line would decline in scenarios 2 and 3 (with or without population growth) compared to scenario 1 (present trend).

**Table 1:** Socioeconomic and ecological indicators at the community level in 2031 in the scenarios considered

	Scenario 1	Scenario 2A	Scenario 2B	Scenario 3A	Scenario 3B
*Socioeconomic impact*					
Mean income	Decreases	Decreases	Increases	Decreases	Stable
Mean above poverty line?	No	No	Yes	No	Yes
% households below extreme poverty line	30%	16%	4%	24%	1%
*Environmental impact*					
Mean secondary forest plot (ha)	2.55	3.89	3.89	3.66	3.66
Chemical inputs*	High	Medium	Low	Medium	Low
Soil degradation**	High	Low	Low	Low	Low

^*^Chemical inputs include NPK fertilizers and use of herbicide

^**^Soil degradation considers soil compacting

Scenario 1 represents a continuation of the observed trend in 2015–2019, Scenario 2 represents the introduction of off-farm employment, with (2a) and without (2b) population growth, scenario 3 represents the introduction of a new conservation policy, with (3a) and without (3b) population growth

Considering the mean size of forest plots in each livelihood type (see table TS7 in Supplementary Material), the proposed policies in scenarios 2 and 3 would help counteract forest fragmentation by increasing the average size of forest plots (Table 1). In terms of landscape pollution, the two proposed policies would be beneficial in that they have a smaller area allocated to pastures, which entail an important load of NPK fertilizers and herbicides (as well as the rearing of non-endemic species) (see Table 1). Note that traditional corn cultivation in the area uses only half the chemical input of cattle rearing (Alonso-Vázquez et al., 2012). The soil degradation indicator reflects the effect of cattle grazing on soil and would also improve in scenarios 2 and 3 with the discouragement of ranching.

## Discussion

### Interpretation of findings

Our first scenario represents a continuation of the observed trend of land use change and population growth in San Isidro in 2015–2019 and hence, expectedly, the results of the simulation are consistent with the expansion of the agricultural frontier and the extensification of cattle ranching observed in south-east Mexico and elsewhere in Latin America (Berget et al., 2021; Cano Castellanos, 2013; Carabias et al., 2015; Rodríguez de Francisco & Budds, 2015). The high investment of labor and inputs required for the traditional crop cultivation in the study region, such as corn (milpa), oil palm or rubber trees encourages farmers to increasingly rely on extensive cattle ranching (Alonso-Vázquez et al., 2012). Cattle ranching, however, is not a traditional livelihood in San Isidro. It was introduced with the colonization of the territory from the 1970s onwards and grazing weeds and practices used elsewhere were adopted without considering the specificities of the local ecosystem (de Vos, 2002). The low productivity of these weed species further contributes to the expansion of the extensive grazing model (Carabias et al., 2015). Nonetheless, given that corn yields in San Isidro are relatively high near its main river, it may be expected that corn cultivation will be kept in these zones, while cattle grazing will expand on the more marginal areas where it is more cost effective than corn cultivation (Alonso-Vázquez et al., 2012; Berget et al., 2021).

In scenario 2, we studied the effect of the availability of off-farm jobs on forest conversion, with and without population growth. Our findings indicate that this policy could increase the average size of forest plots in the community but would not necessarily improve households' economic welfare. It would do so only if there is no population growth. However, the arrival of local off-farm jobs does not seem like a plausible hypothesis in San Isidro. The entire municipality of Marqués de Comillas lacks roads and reliable communication infrastructure (Cano Castellanos, 2013; de Vos, 2002) which compromises the arrival of (agro)industries or other labor intensive economic sectors in the short or medium term. Changes in farming practices and hence in the original profile of the livelihood types may therefore be needed, such as investments in machinery, to increase the economic return of on-farm activities, or investments in infrastructures to realize the option of distant off-farm labor. Otherwise, in the absence of other solutions, migration to large cities has to be expected.

In scenario 3, we analyzed the effects of the adoption of a new conservation scheme to counteract forest conversion to agricultural and ranching land on individually held land. This scenario posits a shift of paradigm in the existing policy, with the goal of forest conservation being prioritized over agriculture and livestock rearing. This policy would also improve forest conservation, but, as we found in scenario 2, the socioeconomic conditions of the households would only improve in the absence of further population growth. Note that the switching of livelihood type from Ct to Ft observed in this scenario does not necessarily mean that *milpa* farming would be abandoned altogether. As per our definition of the livelihood types, the households belonging to the Ft still dedicate a mean surface of 4.5 ha to corn cultivation. Given the relatively isolated location of the community, maintaining the milpa tradition to meet local needs (self-consumption) will remain important.

Scenario 3 is more plausible than scenario 2, given the government’s willingness to change incentive structures for forest conservation through public policy. Such incentive structures are generally easier to implement than those required for stimulating industrial development or other non-farm activities in remote areas. Indeed, in 2020, the federal government started implementing “Sembrando Vida” (Secretaria de Bienestar, 2021) a pilot agroforestry program granting eligible farmers 5000 MXN/month. (As this program took effect after the observed baseline period of our study, it has not been examined here.) Furthermore, the policy suggested in our scenario would not imply additional government spending if it replaced the subsidies given in 2019 for corn cultivation and cattle ranching. Mexican agricultural subsidies have been deemed negative for forest conservation outside protected areas since they have encouraged agricultural and livestock expansion (Moffette et al., 2021).

Our study suggests that the ongoing changes in local livelihoods and land uses are contributing to deforestation in San Isidro and beyond. If farmers continue betting on cattle ranching at the expense of forests, at the current rate of population growth, the social and economic wellbeing of the community is also expected to worsen in the coming years. This will compromise any prospect of local and regional sustainability. Based on our scenario analysis, either creating off-farm job opportunities or implementing forest conservation policies on individually held land could halt or even reverse this trend and increase the forest area, as well as reduce the current trend of soil degradation and agricultural input use. However, any beneficial effect of these policies would depend on a discontinuation of the current trend of population growth. A further decline in the per capita land availability will inevitably put agricultural yields (and, hence, ecological indicators) under pressure to meet local food demand and offset the reduction of income per capita.

### Strengths and shortcomings of the study

The strength of our approach lies in its ability to quantify the entanglement over the relations between livelihood heterogeneity and land use dynamics across hierarchical levels and analytical dimensions. The goal of the scenario analysis informed by archetypes and MuSIASEM is to generate a semantically open framework for the representation of the performance of social-ecological systems in response to trends and shifting policies, incorporating, when and where possible, locally relevant constraints and indicators. For example, in the three scenarios presented, we gave priority to household income as key performance indicator, and we considered land access a key constraint for rural livelihoods. However, other criteria and other constraints could have been considered. In addition, the analysis can potentially be extended to other, higher hierarchical levels of analysis, provided that the livelihood archetypes remain valid.

Although four years (2015–2019) is arguably not a sufficient time lapse to be indicative of a future trend in the studied community, we used this time frame to test our approach for exploring the entanglement of the expected changes in land use in response to conservation strategies with the diversity of livelihood strategies. We underscore that the scenarios analyzed — off-farm employment and a forest conservation program on individually held land—are not conceived to feed a predictive model but rather as an intermediary object (Latour, 1994), useful to structure discussions and involve social actors in an informed deliberation. The proposed method of analysis does not pretend to deliver exact explanations of causal relations nor reliable predictions about the future but aims at providing an analytical framework to support deliberation about the future of a given socio-ecological system, by identifying the relevant factors associated with its sustainability (Berkes & Folke, 1998; Ostrom, 2009). Even though the involvement of local actors in the framing of the analysis did not take place in the present study because of the Covid-19 pandemic, it is evident how participatory processes can boost the robustness and usefulness of our approach.

We acknowledge at least four limitations in the scenarios drawn in this article. First, the quantitative assessments put forward are affected by large uncertainties—i.e., it is not possible to foresee a price trend for agricultural products, since they have been fluctuating between 2015 and 2019 and there is not an identifiable established trend. Second, the 4-year trend analyzed might not capture other socio-ecological dynamics that cannot be observed with the data employed to characterize such a short time span. Third, our study cannot integrate the eventual commercial exchanges of land between community members, which may change household’s land endowments in unpredictable ways. Finally, the scenarios developed do not consider unexpected environmental, social and economic shocks—e.g., climate hazards, migration to urban centers, or arrival of seasonal workers. These could alter the set of fund-flow relations considered in our methodological approach. Overall, these limitations indicate that, in future research, it is essential to rely more on participatory processes to check both the choice of the framing (Giampietro & Bukkens, 2015; Saltelli & Giampietro, 2017) and the reliability of the characterizations, in an iterative manner.

## Conclusions

We have shown that it is critical to account for the heterogeneity of farming systems and livelihood types when designing forest conservation policies, and particularly to understand how such policies may interact with each other and with farmers’ option space, and in turn result in conservation and development trade-offs.

We have demonstrated the key importance of the variable population. If the current trend of population growth continues and community dependence on agriculture persists, land will become scarce in all scenarios analyzed and, in the absence of changes in external conditions, forest conservation will become a “mission impossible.”

Our results show that the inclusion of economic factors in the analysis of forest conservation and conversion matters. Livelihood types facilitate this integration as a transversal construct that allows to (i) connect the biophysical characteristics of the landscape with the socioeconomic characteristics of the community and their reciprocal implications and (ii) to describe the heterogeneity of livelihoods among households.

Finally, we have pointed out that it is necessary to involve local actors in the framing of the analysis. Co-production of information with these actors can help equip a community with the skills required to discuss the expected trade-offs of policy options and make better informed decisions.

## Supplementary Information

Below is the link to the electronic supplementary material.

Supplementary file1 (DOCX 289 KB)

Supplementary file2 (JPG 176 KB)

## Data Availability

All data generated or analyzed during this study are included in this published article.

## Footnotes

1 The PROCEDE Programme (Programa de Certificación de Derechos Ejidales y Titulación de Solares Urbanos), implemented since 1993, aimed at tackling boundary conflicts. It involves legal recognition of the informal plotting internally established within the ejido and thus the registration of the internal land arrangements previously legitimized by the ejido assembly (Braña-Varela & Martínez-Cruz, 2005).

2 21,23 Mexican pesos (MXN) were the equivalent of 1 euro (€) and 18,9 MXN were the equivalent of 1 USD on December 31, 2019 (https://currencies.zone/historic/us-dollar/mexican-peso/december-2019).

3 1 dollar = 14.97 MXN on December 31, 2015 (exchangerates.org.uk).
