# Supplementary material for: Advancing the understanding of forest conservation dynamics through livelihood and landscape change scenarios: a case study in Chiapas, Mexico
Source: Environ Dev Sustain. 2023 Feb 24:1–23. Online ahead of print. doi: 10.1007/s10668-023-02965-z (PMC9951147; doi:10.1007/s10668-023-02965-z)
Supplement: Supplementary file 1 — Supplementary file1 (DOCX 289 KB) [file 10668_2023_2965_MOESM1_ESM.docx]

**Advancing the understanding of forest conservation dynamics through livelihood and landscape change scenarios: a case study in Chiapas, Mexico**

# SUPPLEMENTARY MATERIAL


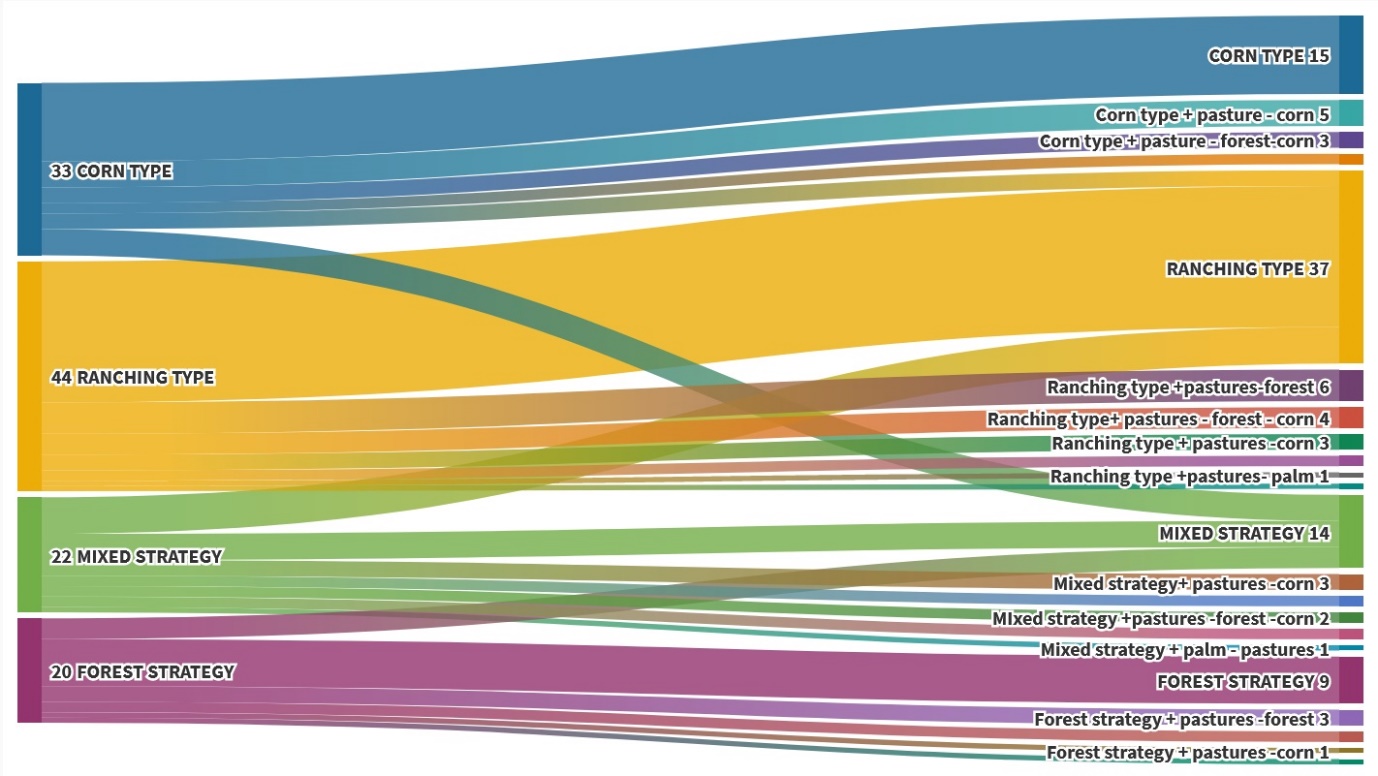


*Fig S1: Land use changes in the households of San Isidro between 2015 and 2019 sorted by livelihood type. The left side represents the distribution in 2015, and the right side the distribution in 2019: some households switch to other types while some others adjust the surfaces of their land use not drastically enough to switch to another type.*

*Table TS1: Land use areas (LU), tons of produce and expected income from agriculture and other sources (subsidies) for each livelihood type in San Isidro. Data refer to the year 2015. Data sources are described in section 2.4.*

| **Corn type** | **LU (ha)** | **Produce (t)** | **Income (MXN)** |
| --- | --- | --- | --- |
| ranching | 3.0 | 2.4 | 72000 |
| corn | 3.5 | 9.7 | 14700 |
| beans | 1.7 | 1.3 | 5500 |
| palm | 0.0 | 0.0 | 0 |
| forest | 3.0 | 0.0 | 0.00 |
| Progan |  |  | 1800 |
| Procampo |  |  | 2300 |
| Prospera |  |  | 13000 |
| Total subsidies |  |  | 17000 |
|  |  |  |  |
| **Ranching type** | **LU (ha)** | **Produce (t)** | **Income (MXN)** |
| ranching | 17.0 | 13.6 | 408000 |
| corn | 1.9 | 5.2 | 8000 |
| beans | 0.9 | 0.7 | 3000 |
| palm | 0.0 | 0.0 | 0 |
| forest | 2.1 | 0.0 |  |
| Progan |  |  | 11000 |
| Procampo |  |  | 4400 |
| Prospera |  |  | 13000 |
| Total subsidies |  |  | 28400 |
|  |  |  |  |
| **Mixed type** | **LU (ha)** | **Produce(t)** | **Income (MXN)** |
| ranching | 8.0 | 6.4 | 192000 |
| corn | 2.6 | 7.4 | 11300 |
| beans | 1.3 | 1.0 | 4200 |
| palm | 1.0 | 2.6 | 3500 |
| forest | 6.5 | 0.0 |  |
| Progan |  |  | 4800 |
| Procampo |  |  | 6200 |
| Prospera |  |  | 13000 |
| Total subsidies |  |  | 24000 |
|  |  |  |  |
| **Forest type** | **LU (ha)** | **Produce(t)** | **Income (MXN)** |
| ranching | 1.0 | 0.8 | 24000 |
| corn | 1.5 | 4.2 | 6375 |
| beans | 0.7 | 0.5 | 2300 |
| palm | 0.0 | 0.0 | 0 |
| forest | 11.0 | 0.0 |  |
| Progan |  |  | 600 |
| Procampo |  |  | 1200 |
| Prospera |  |  | 13000 |
| Total subsidies |  |  | 14800 |
| off income |  |  | 70000 |

*Tab TS2: Projections of socio-economic indicators in San Isidro*

|  | **MXN/person** | | | | |
| --- | --- | --- | --- | --- | --- |
| **YEAR** | **2015** | **2019** | **2023** | **2027** | **2031** |
| Canasta alimentaria ($/person/year) | 10800 | 13200 | 16200 | 18600 | 21000 |
| Canasta total ($/person/year) | 19200 | 23832 | 28800 | 33600 | 38400 |
| S.Isidro population-stagnating | 687 | 814 | 814 | 814 | 814 |
| Members/household-stagnating pop | 5.77 | 6.84 | 6.84 | 6.84 | 6.84 |
| S. Isidro population-growing | 687 | 814 | 941 | 1104 | 1266 |
| Members/household-growing pop | 5.77 | 6.84 | 7.91 | 9.28 | 10.64 |
| ha/person Rt | 4.33 | 3.65 | 3.16 | 2.69 | 2.35 |
| ha/person Mt | 4.16 | 3.51 | 3.04 | 2.59 | 2.26 |
| ha/person Ct | 2.95 | 2.49 | 2.15 | 1.83 | 1.60 |
| ha/person Ft | 2.95 | 2.49 | 2.15 | 1.83 | 1.60 |
|  |  |  |  |  |  |
|  |  |  |  |  |  |

*Tab TS3: Income per capita for every livelihood type in Scenario 1*

| **PRESENT TREND - SCENARIO 1** |  | **MXN/person** | |  |  |
| --- | --- | --- | --- | --- | --- |
|  | **2015** | **2019** | **2023** | **2027** | **2031** |
| Corn type | 16103 | 13583 | 11750 | 10015 | 8734 |
| Ranching type | 72470 | 61130 | 52880 | 45073 | 39305 |
| Mixed strategy | 37135 | 31324 | 27097 | 23096 | 20141 |
| Forest + off farm type | 19064 | 17616 | 15428 | 13312 | 11750 |
| MEAN | 41331 | 37496 | 34503 | 31157 | 28685 |
| Corn type(2015)-> Ranching type | 16103 | 41569 | 35958 | 30649 | 26727 |
| Corn type(2015)->Mixed type | 16103 | 22188 | 19194 | 16360 | 14266 |
| Extreme poverty line (food) | 10800 | 13200 | 16200 | 18600 | 21000 |
| Poverty line (food & basics) | 19200 | 23832 | 28800 | 33600 | 38400 |

*Tab TS4: Income per capita for every livelihood type in Scenario 2*

|  | **MXN/person** | | |  | |  | |  | |  |
| --- | --- | --- | --- | --- | --- | --- | --- | --- | --- | --- |
| **OFF-FARM JOBS - SCENARIO 2a** | |  |  | |  | |  | |  | |
| **growing population** | | **2015** | **2019** | | **2023** | | **2027** | | **2031** | |
| Corn type | | 16103 | 13583 | | 11750 | | 10015 | | 8734 | |
| Ranching type | | 72470 | 61130 | | 52880 | | 45073 | | 39305 | |
| Mixed strategy | | 37135 | 31324 | | 27097 | | 23096 | | 20141 | |
| Forest&off farm job type | | 19954 | 16832 | | 25292 | | 24792 | | 24439 | |
| MEAN | | 41480 | 39174 | | 36134 | | 32511 | | 30019 | |
| Corn type(2015)-> Ranching type | | 16103 | 41569 | | 35958 | | 30649 | | 26727 | |
| Corn type(2015)->Mixed type | | 16103 | 22188 | | 19194 | | 16360 | | 14266 | |
| Extreme poverty line (food) | | 10800 | 13200 | | 16200 | | 18600 | | 21000 | |
| Poverty line (food & basics) | | 19200 | 23832 | | 28800 | | 33600 | | 38400 | |
|  | |  |  | |  | |  | |  | |
| **OFF-FARM JOBS - SCENARIO 2b** | |  |  | |  | |  | |  | |
| **stagnant population** | | **2015** | **2019** | | **2023** | | **2027** | | **2031** | |
| Corn type | | 16103 | 13583 | | 13584 | | 13584 | | 13584 | |
| Ranching type | | 72470 | 61130 | | 61133 | | 61133 | | 61133 | |
| Mixed strategy | | 37135 | 31324 | | 31326 | | 31326 | | 31326 | |
| Forest&off farm job type | | 19954 | 16832 | | 29240 | | 33626 | | 38012 | |
| MEAN | | 41480 | 39174 | | 41774 | | 44096 | | 46691 | |
| Corn type(2015)-> Ranching type | | 16103 | 41569 | | 41571 | | 41571 | | 41571 | |
| Corn type(2015)->Mixed type | | 16103 | 22188 | | 22189 | | 22189 | | 22189 | |
| Extreme poverty line (food) | | 10800 | 13200 | | 16200 | | 18600 | | 21000 | |
| Poverty line (food & basics) | | 19200 | 23832 | | 28800 | | 33600 | | 38400 | |

*Tab TS5: Income per capita for every livelihood type in Scenario 3*

|  | **MXN/person** | |  | |  | |  | |  | |
| --- | --- | --- | --- | --- | --- | --- | --- | --- | --- | --- |
| **CONSERVATION POLICY - SCENARIO 3a** | |  | |  | |  | |  | |  |
| **growing population** | **2015** | **2019** | | **2023** | | **2027** | | **2031** | |  |
| Corn type | 16164 | 13634 | | 11794 | | 10053 | | 8767 | |  |
| Ranching type | 67678 | 57088 | | 49383 | | 42092 | | 36706 | |  |
| Mixed strategy | 37135 | 31324 | | 28999 | | 24717 | | 21554 | |  |
| Forest&off farm job type | 19064 | 17616 | | 21910 | | 18837 | | 16568 | |  |
| MEAN | 39515 | 37327 | | 32860 | | 28494 | | 25272 | |  |
| Extreme poverty line (food) | 10800 | 13200 | | 16200 | | 18600 | | 21000 | |  |
| Poverty line (food & basics) | 19200 | 23832 | | 28800 | | 33600 | | 38400 | |  |
| Forest type | 15840 | 13361 | | 11558 | | 9851 | | 8591 | |  |
|  |  |  | |  | |  | |  | |  |
| **CONSERVATION POLICY - SCENARIO 3b** | |  | |  | |  | |  | |  |
| **stagnant population** | **2015** | **2019** | | **2023** | | **2027** | | **2031** | |  |
| Corn type | 16164 | 13634 | | 13635 | | 13635 | | 13635 | |  |
| Ranching type | 72470 | 57088 | | 57091 | | 57091 | | 57091 | |  |
| Mixed strategy | 37135 | 31324 | | 33525 | | 33525 | | 33525 | |  |
| Forest&off farm job type | 19064 | 17616 | | 25330 | | 25549 | | 25769 | |  |
| MEAN | 39576 | 37456 | | 40000 | | 41106 | | 42227 | |  |
| Extreme poverty line (food) | 10800 | 13200 | | 16200 | | 18600 | | 21000 | |  |
| Poverty line (food & basics) | 19200 | 23832 | | 28800 | | 33600 | | 38400 | |  |
| Forest type | 15840 | 13361 | | 13362 | | 13362 | | 13362 | |  |

*Table TS6: Comparing the public investment in agricultural subsidies (cattle and corn) and that of Scenario 3 policy incentivizing forest conservation.*

| **Year** | **Livelihood types**  **Rt Mt Ct Ft** | | | | | **Total Ag subsidies (MXN)** | **Investment Cons policy (MXN)** |
| --- | --- | --- | --- | --- | --- | --- | --- |
| 2019  (support to corn and cattle) | Subsidy per household (MXN) | 27600 | 24000 | 17650 | 14750 | 2743650 | 0 |
|  | Number of households | 54 | 24 | 25 | 16 | (initial distribution among types) | |
| 2023  (support to conservation) | Subsidy per household (MXN) | 0 | 39000 | 18000 | 66000 | 0 | 2442000 |
|  | Number of households | 54 | 24 | 25 | 16 | (same distribution) | |
| 2027  (support to conservation) | Subsidy per household (MXN) | 0 | 39000 | 18000 | 66000 | 0 | 2718000 |
|  | Number of households | 54 | 28 | 17 | 20 | (expected switching between types) | |
| 2031  (support to conservation) | Subsidy per household (MXN) | 0 | 390000 | 18000 | 66000 | 0 | 2994000 |
|  | Number of households | 54 | 32 | 9 | 24 | (expected switching between types) | |

*Table TS7: Mean sizes of forest plots(ha) in San Isidro(2015) by livelihood type.*

|  | **Ct** | **Rt** | **Mt** | **Ft** |
| --- | --- | --- | --- | --- |
| Mean size of primary forest plots (ha) | 0.98 | 0.44 | 1.98 | 4.72 |
| Mean size of secondary forest plots (ha) | 2.02 | 1.68 | 4.48 | 6.51 |
