# Supplementary figures and images for: Advancing the understanding of forest conservation dynamics through livelihood and landscape change scenarios: a case study in Chiapas, Mexico
Source: Environ Dev Sustain. 2023 Feb 24:1–23. Online ahead of print. doi: 10.1007/s10668-023-02965-z (PMC9951147; doi:10.1007/s10668-023-02965-z)

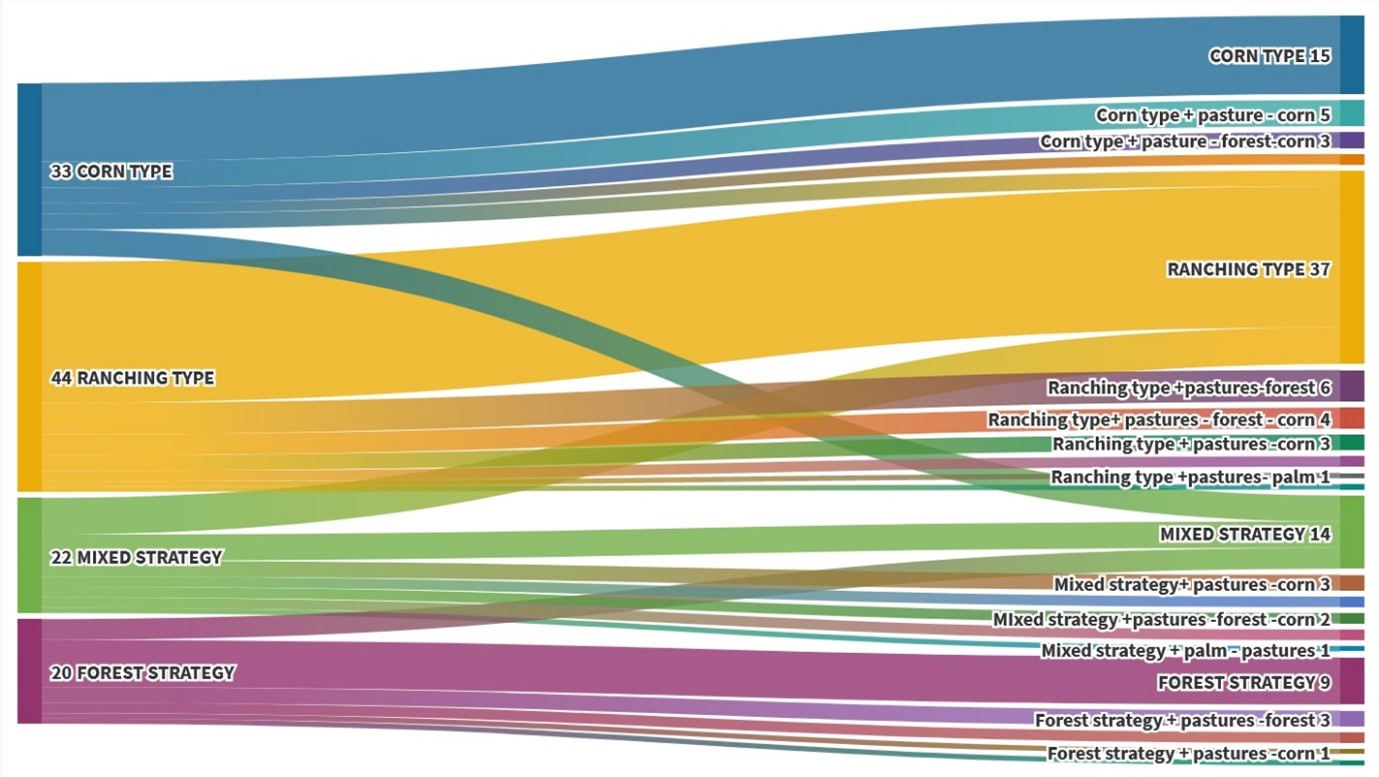

Supplement: Supplementary file 2 — Supplementary file2 (JPG 176 KB) [file 10668_2023_2965_MOESM2_ESM.jpg]
